# Efficient minimizer orders for large values of *k* using minimum decycling sets

**DOI:** 10.1101/gr.277644.123

**Published:** 2023-07

**Authors:** David Pellow, Lianrong Pu, Bariş Ekim, Lior Kotlar, Bonnie Berger, Ron Shamir, Yaron Orenstein

**Affiliations:** 1 Blavatnik School of Computer Science, Tel-Aviv University, Tel Aviv 6997801, Israel;; 2 Computer Science and Artificial Intelligence Laboratory, Massachusetts Institute of Technology, Cambridge, Massachusetts 02139, USA;; 3 Department of Computer Science, Ben-Gurion University of the Negev, Beer-Sheva 8410501, Israel;; 4 Department of Mathematics, Massachusetts Institute of Technology, Cambridge, Massachusetts 02139, USA;; 5 Department of Computer Science, Bar-Ilan University, Ramat-Gan 5290002, Israel;; 6 The Mina and Everard Goodman Faculty of Life Sciences, Bar-Ilan University, Ramat-Gan 5290002, Israel

## Abstract

Minimizers are ubiquitously used in data structures and algorithms for efficient searching, mapping, and indexing of high-throughput DNA sequencing data. Minimizer schemes select a minimum *k*-mer in every *L*-long subsequence of the target sequence, where minimality is with respect to a predefined *k*-mer order. Commonly used minimizer orders select more *k*-mers than necessary and therefore provide limited improvement in runtime and memory usage of downstream analysis tasks. The recently introduced universal *k*-mer hitting sets produce minimizer orders with fewer selected *k*-mers. Generating compact universal *k*-mer hitting sets is currently infeasible for *k* > 13, and thus, they cannot help in the many applications that require minimizer orders for larger *k*. Here, we close the gap of efficient minimizer orders for large values of *k* by introducing *decycling*-*set*-*based minimizer orders*: new minimizer orders based on minimum decycling sets. We show that in practice these new minimizer orders select a number of *k*-mers comparable to that of minimizer orders based on universal *k*-mer hitting sets and can also scale to a larger *k*. Furthermore, we developed a method that computes the minimizers in a sequence on the fly without keeping the *k*-mers of a decycling set in memory. This enables the use of these minimizer orders for any value of *k*. We expect the new orders to improve the runtime and memory usage of algorithms and data structures in high-throughput DNA sequencing analysis.

As the number and depth of high-throughput sequencing experiments grow, efficient methods to map, store, and search DNA sequences have become critical for their analysis. Sequence sketching is a fundamental building block of many of the basic sequence analysis tasks, such as assembly (Ekim et al. 2021; Rautiainen and Marschall 2021), alignment (Li 2018; Dutta et al. 2022; Sahlin 2022), binning (Deorowicz et al. 2015; Chikhi et al. 2016; Flomin et al. 2022), and indexing (Holley and Melsted 2020; Marchet et al. 2021). The common principle in all sketching techniques is the consistent selection of representative *k*-mers from longer DNA sequences for indexing these sequences in data structures or algorithms. A key parameter for evaluating and comparing sketching schemes is density (Marçais et al. 2017), which is defined as the fraction of *k*-mers selected from a sequence by the scheme.

One of the most common sequence sketching techniques is minimizers (Schleimer et al. 2003). The minimizer of an *L*-long sequence is the minimum among all the *k*-mers that the sequence contains, according to some order *o* over the *k*-mers. Selecting the minimizers from all *L*-long windows of a sequence provides a sketch of that sequence. Minimizer schemes provide a window guarantee; that is, one representative *k*-mer is selected from every *L*-long window for any desired value of *L*. Minimizers have low density as the minimum *k*-mer is likely to persist across multiple overlapping windows. However, the commonly used lexicographic and random *k*-mer orders have been shown to have far from optimal density (Marçais et al. 2017).

A recent breakthrough in developing minimizer orders with low density has been achieved by compact universal *k*-mer hitting sets (UHSs) (Orenstein et al. 2017). A UHS is a set of *k*-mers guaranteed to hit (i.e., have a member included in) any *L*-long sequence. In terms of a complete de Bruijn graph (dBG) of order *k*, a minimum UHS is a minimum set of nodes whose removal leaves no path of length *L* − *k* + 1 nodes in the graph. Heuristic algorithms for finding a compact UHS include DOCKS (Orenstein et al. 2017) and PASHA (Ekim et al. 2020), both of which approach UHS construction as a path-covering problem in a complete dBG. Both algorithms first identify a minimum decycling set (MDS), which is a minimum set of *k*-mers guaranteed to hit any infinitely long sequence, and then extend this set into a UHS. An MDS can be generated in time linear in the dBG size (Mykkeltveit 1972).

UHS-based minimizer orders were shown to achieve lower density than common orders (Marçais et al. 2017; Ekim et al. 2020). However, constructing and storing UHSs are inefficient owing to the exponential dependence of the heuristic algorithms on *k*, and currently, compact UHSs are available only for *k* ≤ 13. As a result, UHS-based minimizer orders could not be used in many applications that require larger values of *k*, such as long-read mapping (Li 2018), assembly (Rautiainen and Marschall 2021), and indexing (Holley and Melsted 2020; Pibiri 2022). Moreover, another bottleneck arising in these applications is storing and querying UHS *k*-mers, as the UHS size grows exponentially with *k*.

Partly because of the challenges in constructing UHSs, other recent works focused on developing sequence-specific minimizer orders. For example, sequence-specific minimizer orders were used in binning applications to achieve lower maximum bin size or more balanced bin sizes than general minimizers (Chikhi et al. 2016; Flomin et al. 2022). Hoang et al. (2022) used deep learning to achieve sequence-specific low-density minimizers for much larger *k* (up to 320). Polar-set-based sequence-specific minimizers achieved very low density for *k* ≤ 25 (Zheng et al. 2021). These solutions are tailored to a specific sequence set, and thus, different orders must be generated for every sequence.

In this work, we developed new methods to construct universal, low-density minimizer orders that scale to larger *k*. Motivated by the fact that current algorithms, DOCKS and PASHA, generate a UHS on top of an MDS, we defined minimizer orders based only on an MDS. For most combinations of *k* and *L*, most of the *k*-mers in the UHSs generated by these algorithms are in the MDS. In addition, generating an MDS is the fastest part of UHS construction in these algorithms, taking only Θ(|Σ|^*k*^) for alphabet Σ. Moreover, the same order is used for all *L* and a given *k*, unlike DOCKS and PASHA, which need to compute a UHS for every combination of *k* and *L*. Furthermore, the longest remaining path in a complete dBG after removal of an MDS was recently shown to be bounded by *O*(*k*^3^) (Zheng et al. 2020a), so for large values of *L*, it is likely that many of the windows contain a *k*-mer from an MDS.

As the MDS size grows exponentially in *k*, precomputing, storing, and querying it become infeasible for large *k*. We overcome this limitation by presenting an efficient method to test in *O*(*k*) time if a *k*-mer belongs to the MDS, which enables computing the minimizers in a sequence according to our MDS-based order on the fly. Thus, the minimizer orders that we defined provide, for the first time, universal orders with low density that easily scale to any value of *k* and to any desired window length.

## Methods

### Preliminaries

We begin by providing definitions and theoretical background and describing relevant related work (for further background, see Orenstein et al. 2017; Edgar 2021).

#### Basic definitions

For a string *S* over an alphabet Σ, a *k-mer* is a contiguous substring of length *k*. We denote the *k*-mer starting at position *i* as *S*[*i*, *i* + *k* − 1].

A *k-mer order* is a function on *k*-mers *o*:Σ^*k*^ → R. We say that *k*-mer *x*_1_ is *smaller than x*_2_ under *o* (*x*_1_ < _*o*_*x*_2_) iff *o*(*x*_1_) < *o*(*x*_2_).

A *de Bruijn graph* (dBG) of order *k* is a directed graph in which every node is labeled with a distinct *k*-mer, and there is a directed edge from node *a* to *b* iff the (*k* − 1)-long suffix of *a* is the same as the (*k* − 1)-long prefix of *b*. The edge is labeled with the (*k* + 1)-long merge of the two labels. A *complete dBG* has a node for every possible *k*-mer and an edge for every possible (*k* + 1)-mer. Paths in a dBG of order *k* represent sequences, and a path of *w* nodes represents a sequence of *w* overlapping *k*-mers.

#### Minimizers

A *minimizer scheme* is a function *f*_*k*,*w*,*o*_:Σ^*w*+*k*−1^ → {0, …, *w* − 1}. Function *f* returns the position of the minimum *k*-mer under *o* in a given window of *w* overlapping *k*-mers (i.e., in an *L* = *w* + *k* − 1 long sequence). By convention, ties are broken by choosing the leftmost *k*-mer. The *minimizers* of a string *S*, denoted as $M_{k,w,o}(S)$, are all the positions in the string that are selected by applying the scheme to all overlapping *L*-long windows of *S*.

The *expected density* of a minimizer scheme is the expected fraction of *k*-mer positions that will be selected as minimizers over an infinitely long random i.i.d. sequence. The *particular density* of a minimizer scheme on a specific sequence *S* (e.g., the human genome) is the fraction of *k*-mer positions selected by the scheme on that sequence. The *expected* (*particular*) *density factor* is the expected (particular) density multiplied by (*w* + 1).

The expected density factor of a random minimizer is two (Marçais et al. 2017). Marçais et al. (2018) discuss the asymptotic behavior of minimizer density as *k* → ∞ or *w* → ∞ and prove a general lower bound of $\frac{1.5+\max\left(0,\frac{k-w}{w}\right)+1/\;2w}{w+k}$ for the density of any forward scheme, a class of generalized sketching schemes that includes minimizers.

Given an ordered partition of Σ^*k*^, $\Pi =[C_{1},\ldots ,C_{m}]$, and a *k*-mer order *h*, we say that minimizer order *o*_Π,*h*_ is *compatible with* Π and *h* if for $x_{1}\in C_{i}$, $x_{2}\in C_{j}$, $i<j\Rightarrow x_{1}{<}_{o_{\Pi ,h}}x_{2}$, and if *i* = *j*, then $x_{1}{<}_{o_{\Pi ,h}}x_{2}\Leftrightarrow h(x_{1})<h(x_{2})$. In other words, the partition order determines the order between elements from different sets, and another order (typically random) determines the order within each set.

#### Minimum decycling sets

A *decycling set* of a graph *G* = (*V*, *E*) is a set of nodes whose removal results in an acyclic graph. Finding an MDS (also called feedback vertex set) in an arbitrary graph is NP-hard (Karp 1972). We are interested in an MDS of a complete dBG of order *k*. Mykkeltveit (1972) gave an efficient algorithm to construct such a set, which we denote by $D_{k}$, in time linear in the size of a complete dBG, that is, Θ(|Σ|^*k*^). A *pure cycle* is a set of nodes corresponding to all the cyclic rotations of some *k*-mer (Mykkeltveit 1972). Mykkeltveit showed that $D_{k}$ contains a single node from each pure cycle in a complete dBG. Moreover, each pure cycle defines a conjugacy class, and thus, the pure cycles factor the complete dBG; namely, every *k*-mer belongs to exactly one of the pure cycles.

**Figure 1.**
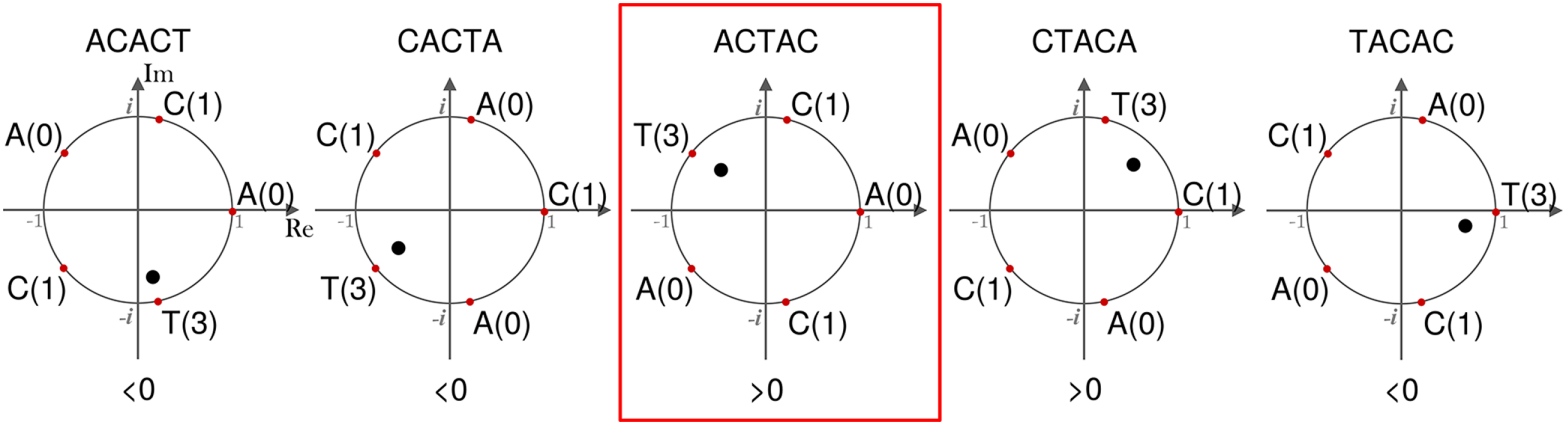
Mykkeltveit embedding. The embedding is shown for the rotations of the *k*-mer ACACT, indicated *above* each plot. Each letter of the *k*-mer corresponds to a weight (in parentheses) placed at the *k*th roots of unity (red dots). The embedding represents the center of mass of the *k*-mer (black dot). The sign of each embedding projected onto the imaginary axis is shown *below* each rotation. In this example, ACTAC (red box) is the first counterclockwise rotation *x* with I(*x*) > 0 and is thus included by Mykkeltveit's algorithm in the MDS.

To determine which of the cyclic rotations of a *k*-mer to include in $D_{k}$, Mykkeltveit defined an *embedding* of *k*-mers as a weighted sum of the *k*th complex roots of one in the complex plane. For a *k*-mer *x*, M(x)=(R(x),I(x))= $\left(\overset{k-1}{\underset{i=0}{\sum }}\;x_{i}\cos\;\left(\frac{2\pi i}{k}\right),\;\overset{k-1}{\underset{i=0}{\sum }}\;x_{i}\sin\;\left(\frac{2\pi i}{k}\right)\right)$, where *x*_*i*_ is the numeric encoding of the *i*th character of *x* (in our case the encoding of the DNA alphabet is A = 0, C = 1, G = 2, T = 3) (Fig. 1). We say a rotation *x* is *positive* (*negative*) if I(*x*) > 0 (<0). All positive (negative) rotations of a *k*-mer are consecutive, and either all rotations have I(*x*) = 0 or the two blocks of consecutive positive and negative rotations are separated by at most one rotation with I(*x*) = 0 on either side (Lemma 1 in Mykkeltveit 1972). The MDS constructed by Mykkeltveit's algorithm includes, for each conjugacy class, the first positive counterclockwise rotation. When all rotations have I(*x*) = 0, any arbitrary *k*-mer from the cycle can be selected.

Mykkeltveit's algorithm has an efficient implementation owing to Knuth (https://www-cs-faculty.stanford.edu/~knuth/programs/unavoidable2.w). This implementation uses the FKM algorithm (Fredricksen and Maiorana 1978) to enumerate the *k*-mer conjugacy classes in lexicographic order. The representative selected for each class is the first positive one, and for classes with I(*x*) = 0 for all *k*-mer rotations, the lexicographically smallest *k*-mer is included in the decycling set. An MDS consists of Θ(|Σ|^*k*^/*k*) *k*-mers.

#### Universal hitting sets

A *universal hitting set* (UHS) $U_{k,L}\subseteq \Sigma^{k}$ is a set of *k*-mers such that any *L*-long string contains at least one *k*-mer from $U_{k,L}$ as a contiguous substring. By construction, at least one *k*-mer from $U_{k,L}$ must appear in every window of *w* = *L* − *k* + 1 overlapping *k*-mers, and thus, the nodes represented by $U_{k,L}$ will be a covering set for all *w*-long paths in a complete dBG of order *k* (Marçais et al. 2017).

Two algorithms proposed for UHS construction are DOCKS (Orenstein et al. 2017) and PASHA (Ekim et al. 2020). They first generate an MDS using Mykkeltveit's algorithm. Both algorithms then add *k*-mers greedily until no path of length *w* remains in the graph. DOCKS uses dynamic programming to compute the number of *w*-long paths covered by each node in the remaining graph, resulting in a runtime of *O*((1 + *p*)|Σ|^*k*+1^*L*) for *p* iterations of node removal. Using DOCKS, small UHSs have been constructed only for *k* ≤ 11. Minimizer orders compatible with these UHSs were shown to have lower density than random orders (Marçais et al. 2017).

PASHA uses a randomized approximation algorithm for Set Cover to remove a small number of nodes in the remaining graph, resulting in a runtime of $O(\left(L^{2}|\Sigma {|}^{k+1}\log^{2}(|\Sigma {|}^{k}))/(\epsilon \delta^{3}\right))$, where δ and ϵ are parameters of the approximation guarantee. UHSs have been constructed using PASHA only for *k* ≤ 13, and the density of minimizer orders compatible with them was observed to be slightly higher than that of minimizer orders compatible with DOCKS UHSs.

Miniception (Zheng et al. 2020b) constructs a UHS for parameters *k* and *L* with an additional parameter *k*_0_ < *k*. *k*-mers with the first or the last *k*_0_-mer as their minimizers are added into the UHS. This set does not need to be precomputed, and instead, membership in the set can be determined on the fly for each *k*-mer in a sequence. Minimizer orders compatible with Miniception-generated UHSs can therefore be computed efficiently for any *k* but were shown to have higher density than orders compatible with UHSs constructed by PASHA.

### Decycling-set-based minimizer orders

Given an MDS $D_{k}$, we define a *k*-mer order in which *k*-mers in $D_{k}$ precede all other *k*-mers, and for pairs not determined by this rule, the order is random. Formally, we define the ordered partition $\Pi =(D_{k},\Sigma^{k}\setminus D_{k})$ and a pseudorandom *k*-mer order *h* and use a minimizer order compatible with Π and *h*. We implement a pseudorandom order efficiently by XOR-ing the binary representation of a *k*-mer with a random 2*k*-bit seed as was performed by Wood and Salzberg (2014) and Flomin et al. (2022). $D_{k}$ can be constructed efficiently using Knuth's implementation of Mykkeltveit's algorithm (Mykkeltveit 1972) as described above.

**Figure 2.**
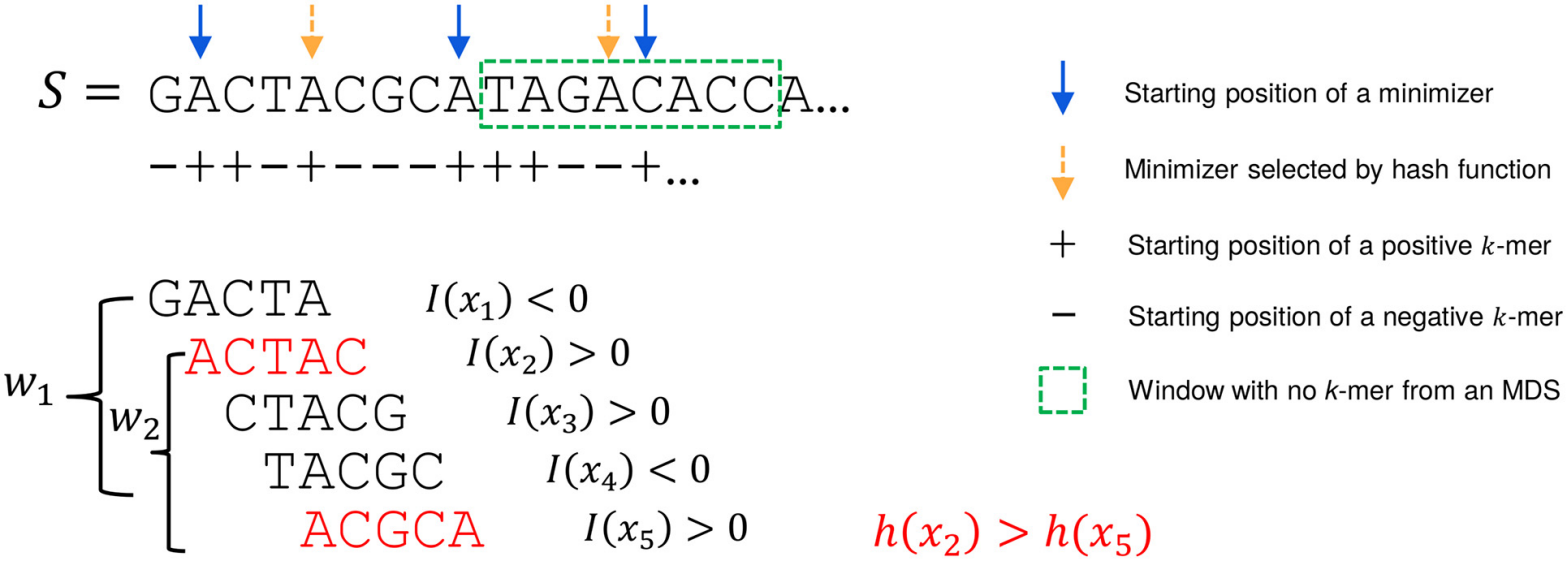
Decycling-set-compatible minimizers. An example of selecting minimizers based on an MDS with parameters *k* = 5, *L* = 8. *k*-mers in the *leftmost* two windows are shown *below* the sequence, with *k*-mers in the decycling set in red. The second window *w*_2_ contains two *k*-mers from the decycling set, and a hash function (lexicographic order in this example) is used to select ACGCA as the minimizer. The sequence boxed in green is a window with no *k*-mer from the decycling set, and thus, the lexicographically smallest *k*-mer is selected as the minimizer by the hash function.

For large values of *k*, when $D_{k}$ is too large to store or takes too much time to compute, we instead scan the target sequence and, for every *k*-mer, test its membership in $D_{k}$ on the fly using the procedure outlined in Algorithm 1 (see also Fig. 2). The imaginary parts of the embeddings of a *k*-mer *x* and its clockwise rotation *x*′ are computed in *O*(*k*) time and compared to determine if *x* is the first positive counterclockwise rotation. If I(*x*) = I(*x*′) = 0, then the algorithm determines whether *x* is a lexicographically smallest rotation in *O*(*k*) time.

Proposition 1 **(Algorithm 1 correctness).**

*Algorithm 1 correctly determines whether a k-mer is a member of* $D_{k}$ *in time O(k)*.

Algorithm 1: **Minimum-decycling-set membership**

*Input: k*-mer *x*

*Output:* Membership in the MDS $D_{k}$

1: **for** *i* ∈ [0, *k* − 1] **do** *c*_*i*_ = sin(2π*i*/*k*)

2: $I(x)=\overset{k-1}{\underset{i=0}{\sum }}\;c_{i}x_{i}$

3: *x*′ = *x*_*k*−1_*x*_0_*x*_1_..*x*_*k*−2_

4: $I(x^{\prime })=\overset{k-1}{\underset{i=0}{\sum }}\;c_{i}x_{i}^{\prime }$

5: **if** I(*x*) > 0 **then** ▹ Check if *x* is the first rotation with I(*x*) > 0

6:  **if** I(*x*′) ≤ 0 **then** return true

7: **else if** I(*x*) = 0 **then**

8:  **if** I(*x*′) = 0 **then** ▹ Check if *x* is the lexico. smallest rotation

9:   *i* ← 0

10:  ** for** *j* ∈ [1, 2*k* − 1] **do**

11:    **if** $x_{j\;\mathrm{mod}\;k}<x_{i}$ **then** return false

12:    **if** $x_{j\;\mathrm{mod}\;k}>x_{i}$ **then** *i* = 0

13:    **else** *i* ← *i* + 1

14:    **if** (j≥k−1)∧(imodk=0) **then** return true

15: return false

Proof. The proof follows from the definition of $D_{k}$. We say that a *k*-mer *x* is *positive, negative,* or *nonpositive* if I(*x*) > 0, < 0, or ≤0, respectively. Recall that a *k*-mer $x\in D_{k}$ iff either: (1) it is the first positive counterclockwise rotation in its conjugacy class or (2) all *k*-mers in the conjugacy class have I(*x*) = 0 and *x* is a lexicographically smallest rotation.

For 1 above, line 6 returns true iff the input *k*-mer *x* is the first positive counterclockwise rotation in its conjugacy class; that is, *x* has I(*x*) > 0, and the one-letter clockwise rotation of *x*, denoted *x*′, has I(*x*′) ≤ 0.

For 2 above, note that if two consecutive rotations of a *k*-mer *x*, *x*′ have I(*x*) = I(*x*′) = 0 (lines 7–8), then all *k*-mers in that conjugacy class have zero embedding by Lemma 1 from Mykkeltveit (1972). The loop in lines 10–14 checks all possible rotations of *x* and returns false if there is a *k*-mer that is lexicographically smaller than *x* (line 11). Otherwise, it returns true if either all possible rotations are lexicographically larger than *x* (in this case, *i* = 0 and *j* = *k* − 1) or *x* is a lexicographically smallest rotation but is identical to another rotation (in this case, *i* reaches *k* and *j* ≥ *k* − 1; line 14).

The embedding computations (lines 1, 2, and 4) take *O*(*k*) time. The loop beginning on line 10 can run for at most 2*k* times and performs constant time computations per iteration for a total running time of *O*(*k*).

### Double decycling-set-based minimizer orders

By symmetry, Mykkeltveit's construction can be used to create an MDS using the first counterclockwise negative *k*-mer *x* in each conjugacy class rather than the first positive one. We refer to this set as the *symmetric* decycling set ${\tilde{D}}_{k}$. The decycling set and symmetric decycling set divide sequences according to the following interesting property:

Theorem 1 (remaining path partition). *In any remaining path in a complete dBG after removing* $D_{k}$*, all the positive nodes (i.e., labeling positive k-mers) precede all the nonpositive nodes*.

In other words, a remaining path must consist of two distinct parts: a *positive part*, containing only positive *k*-mers, followed by a *nonpositive part* consisting of only nonpositive *k*-mers. The proof relies on two lemmas:

Lemma 1. *The k-mers associated with all incoming neighbors of a node x in a dBG have the same* I(*x*).

Proof. All incoming neighbors *y* of *x* differ only in *y*_0_ and have embedding with $I(y)=y_{0}\sin\;(0)+\overset{k-1}{\underset{i=1}{\sum }}\;\sin\;(2\pi i/k)y_{i}=\overset{k-1}{\underset{i=1}{\sum }}\;\sin\;(2\pi i/k)y_{i}$.

Lemma 2. *The pure cycles factor a complete dBG; namely, every k-mer belongs to exactly one of the pure cycles*.

Proof. Every *k*-mer is on some pure cycle corresponding to its rotations. Assume the contrary that *k*-mer *x* is on two distinct pure cycles, *C*_1_ and *C*_2_. Let *y* be the last common node in the path in $C_{1}\cap C_{2}$ starting from *x*. Then, the edges out of *y* in the two cycles are distinct, contradicting the fact that both correspond to the cyclic rotation of *y*.

Proof (Theorem 1). Let *x*_*i*_ be the first nonpositive node in a remaining path *x*_1_, …, *x*_*t*_ and assume the contrary that there exists a positive *x*_*j*_ for *j* > *i*. W.l.o.g. assume *x*_*j*_ is the first with that property in the path. Let *C* be the pure cycle that contains *x*_*j*_. *C* exists, and it is well defined by Lemma 2. Let *y* be the node preceding *x*_*j*_ in *C*. By Lemma 1, I(*x*_*j*−1_) = I(*y*). Because *y* is nonpositive, *x*_*j*_ should be in $D_{k}$ as the first positive node in *C*, a contradiction.

By a similar argument, in a remaining path after removing ${\tilde{D}}_{k}$, the negative nodes precede all other nodes. Thus, removal of a *double decycling set* consisting of $D_{k}\cup {\tilde{D}}_{k}$ would leave only short remaining paths that cannot contain both negative and positive *k*-mers.

We define a partition-compatible minimizer order based on double decycling sets with $\Pi =\{D_{k},{\tilde{D}}_{k}\setminus D_{k},\Sigma^{k}\setminus (D_{k}\cup {\tilde{D}}_{k})\}$. Because the double decycling set leaves even shorter remaining paths, we hypothesize that this minimizer order will achieve lower density compared with the one using only a single decycling set.

## Results

We compared the performance of our new MDS-based minimizer orders to random orders and to orders based on DOCKS, PASHA, and Miniception (Zheng et al. 2020b), across a range of *k* and *L* values. Miniception uses lexicographic order by default, which was shown to have worse performance than random order. To be fair, here we modified Miniception to use the same random hash as our implementations and ran it with the recommended parameters.

We evaluated performance on expected and particular density factors. We estimated expected density factors by calculating density on 10 random i.i.d. sequences of 10 million nucleotides, each time with a different pseudorandom seed for the *k*-mer order. We calculated particular density factors on 10 randomly selected 10 million–nt segments from Chromosome X of the CHM13 telomere-to-telomere human genome assembly (Nurk et al. 2022) using different pseudorandom seeds. Particular densities for more real genomic sequences are also presented in the Supplemental Figures.

### MDS-based orders outperform UHS-based orders

**Figure 3.**
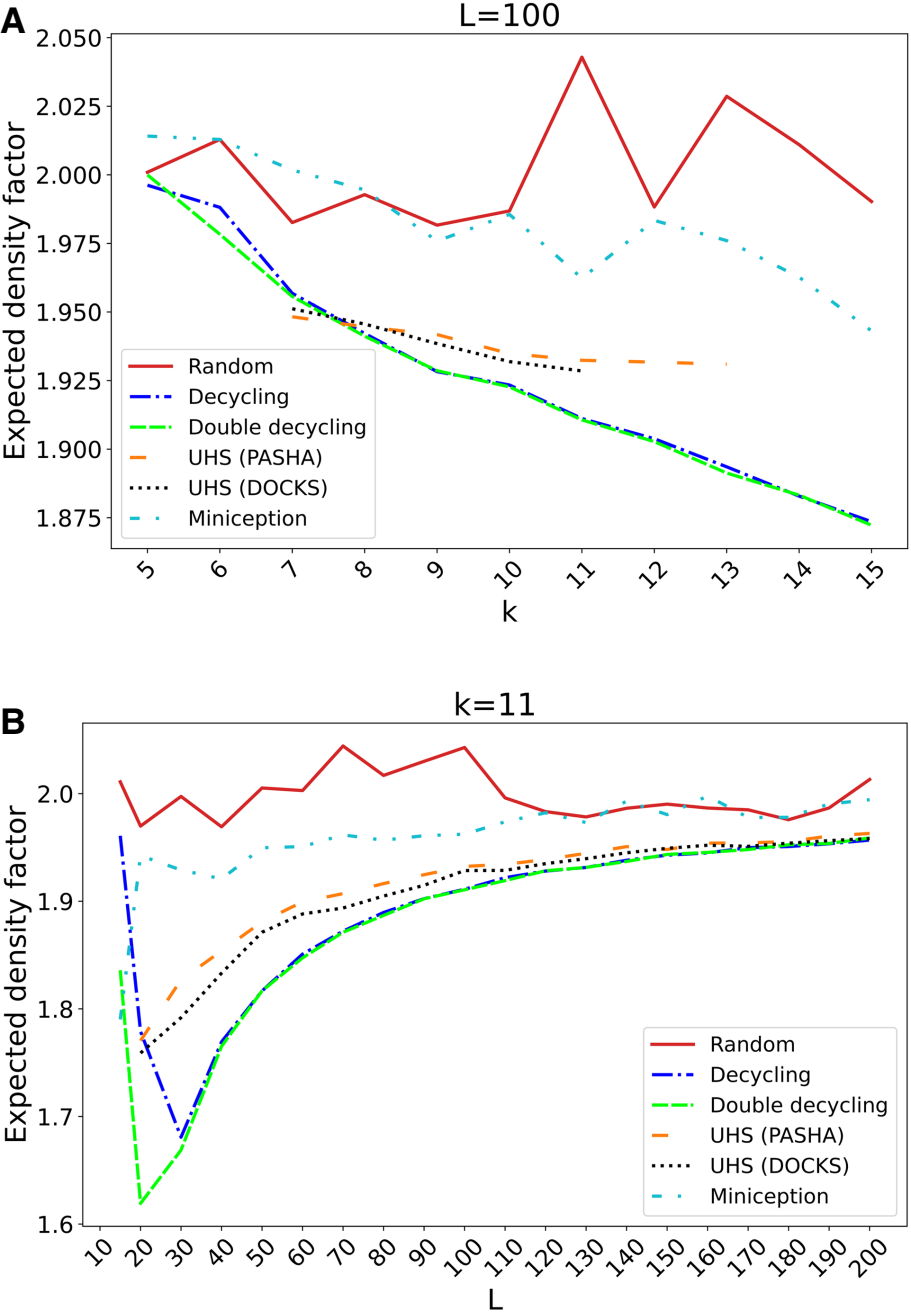
Expected density factors of various minimizer orders. The expected density factors of various minimizer orders are compared for *L* = 100 and varying 5 ≤ *k* ≤ 15 (*A*) and for *k* = 11 and 15 ≤ *L* ≤ 200 (*B*).

The expected density factors of the tested orders are compared for 5 ≤ *k* ≤ 15 and *L* = 100 in Figure 3A and for *k* = 11 and 10 ≤ *L* ≤ 200 in Figure 3B. Average density factors over the runs are shown without error bars for visual clarity. The same plots with error bars and theoretical lower bounds are in Supplemental Figure S1.

UHSs for *k* < 7 and *L* = 100 consist only of an MDS and thus are not shown. DOCKS-generated UHSs were computed for *k* up to 11 and PASHA-generated UHSs for *k* up to 13. UHSs for larger *k* could not be generated owing to the runtime and storage required for every combination of *k* and *L*. In contrast, our new MDS-based orders have the distinct advantage of being easily computable on the fly for any (*k*, *L*) combination.

The MDS-based orders consistently perform similarly or better than UHS-based orders. As hypothesized, the double decycling-set order has lower density than the decycling-set order. Miniception performs better than random and worse than the UHS-based orders, as was previously shown (Zheng et al. 2020b). As expected, random orders typically perform worst across most of the range of *L*, and the relative improvement of UHS- and MDS-based orders compared with random orders increases with *k*. Conversely, as *L* grows for fixed *k*, the density factors of the different methods tend to perform similarly to random because longer windows are more likely to contain multiple *k*-mers from the sets defining the order (UHS or MDS), and the *k*-mers within the set are ordered randomly. MDS-based orders have much lower standard errors than the others, likely because the decycling-set *k*-mers remain the same for all repeated runs regardless of the random seed.

The particular density factors are presented in Supplemental Figures S1 and S2 as they display a similar trend as the expected factors. The particular density is slightly higher and more variable, with higher standard errors, as it is dependent on the particular sequence, and *k*-mer usage is not uniformly distributed in real genomic sequences. However, the overall shape of the density curves and the performance ranking among the methods remain the same.

### Scaling MDS-based orders to *k* ≥ 20

**Figure 4.**
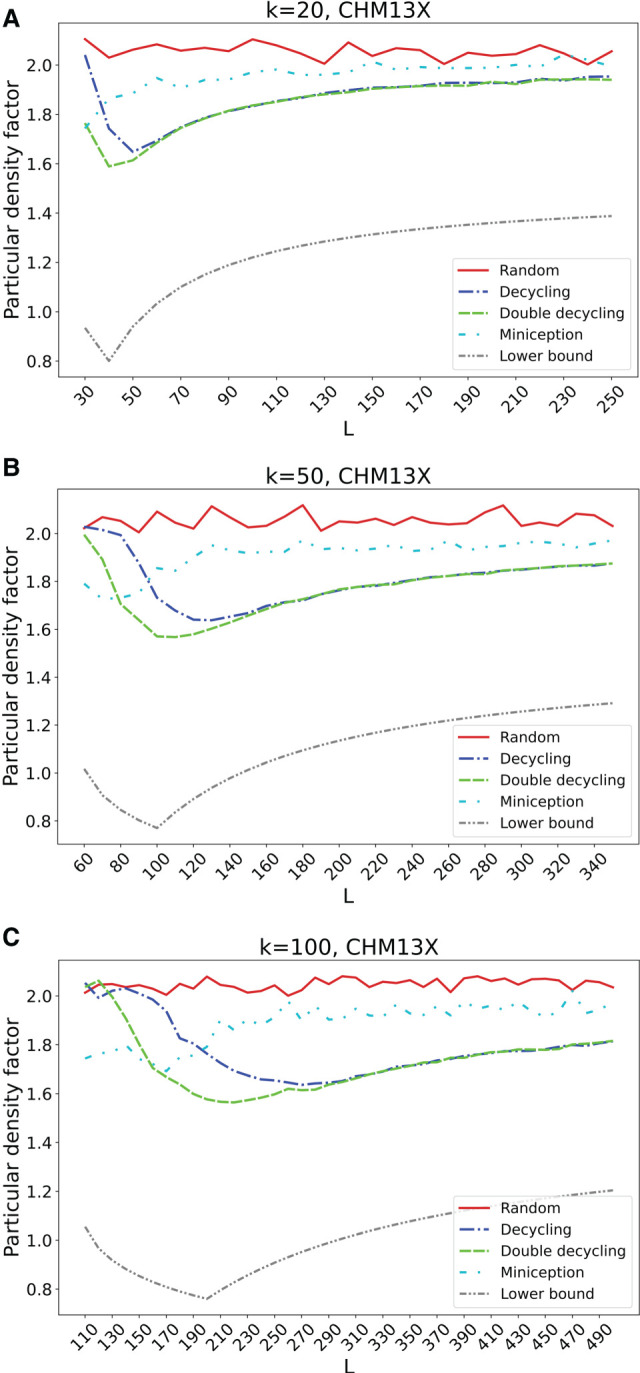
Particular density factors of minimizer orders for large *k*. The particular density factors computed on 10 million–nt samples from the X Chromosome of the CHM13 assembly of various minimizer orders are compared for *k* = 20 (*A*), *k* = 50 (*B*), and *k* = 100 (*C*) over variable *L* values. The theoretical lower bound from Marçais et al. (2018) is also shown for comparison.

We compared the MDS-based orders to the random baseline order and to Miniception for values of *k* greater than those that are feasible to run with DOCKS and PASHA. Figure 4, A through C, shows particular density factors for *k* = 20, 50, and 100 on random segments of the human X Chromosome. Average density factors over the repeated runs are shown without error bars for visual clarity. The same plots with error bars as well as the expected density factors for *k* = 20, 50, and 100 are in Supplemental Figure S1, and particular density factors of more genome sequences are in Supplemental Figure S2.

As *k* grows, the advantage of the MDS-based orders becomes even more pronounced, and the double decycling-set-based order improves more significantly over the decycling-set-based order. This is true in particular for smaller *L*, with the differences between the decycling-set-based and double decycling-set-based orders beginning to diminish as *L* > 2*k*. The advantage compared to Miniception is maintained for large values of *k* across most *L* values, and the MDS-based minimizer orders achieve density factors that are up to 20% lower than Miniception-based orders. Miniception-based orders achieve lower density for small *L* as MDS-based orders are similar to random in that regime because most windows do not contain a member of the MDS. In contrast, Miniception approaches its theoretical density factor of 1.67 while *L* ≤ 2*k* and converges to (but remains below) the performance of random for larger *L*. In most cases, the particular density factor is slightly higher than the expected density factor, but the overall shape of the performance curves and the relative performance of the methods are the same for both particular and expected density.

There exists a consistent gap between the theoretical lower bound for forward schemes, which is not known to be tight for minimizers and converges to 1.5 as *L* increases, whereas the MDS-based orders converge to two. Interestingly, the shape of the density factor curves is similar to the lower bound with a minimum around *L* = 2*k*.

### Runtime and memory usage

We report runtime measurements under three regimes of our MDS-compatible minimizer scheme implementations. For *k* < 20, the MDS is precomputed and stored as a boolean vector. Thus, *k*-mer set membership is determined with an *O*(1) lookup. The exponential growth of the MDS makes it impossible to precompute or store in memory for even moderately larger *k*. Instead, we compute *k*-mer set membership on the fly for every *k*-mer in *O*(*k*) time using Algorithm 1. As *k*-mers are represented using two bits per nucleotide, our implementation in C++ can use CPU-supported 128-bit operations for *k* ≤ 63 (an additional bit of the representation is used to indicate set membership). We used the GMP library (https://gmplib.org/) to support operations for *k* > 63. As a result, the process is an order of magnitude slower than CPU-supported operations. We report runtimes to compute minimizers of a random 10 million–nt sequence for all methods and three values of *k* representing the different regimes in Table 1. Runtimes were measured on a 44-core, 2.2-GHz server with 792 GB of RAM, using a single thread. We report averages and standard deviations over 10 runs.

**Table 1.**
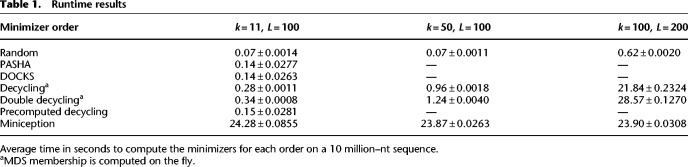
Runtime results

We implemented the pseudorandom order by a simple XOR operation and a comparison of the resulting integers. Thus, the random order is extremely fast for all *k* and *L* values. The lookup and comparison used by the precomputed decycling set and UHS methods are also fast but limited to relatively small *k*. Algorithm 1 with the more complex embedding calculation and comparison is slower, and the runtime increases with *k*. Our implementation's runtime increases by one to two orders of magnitude for *k* > 63. However, even the slowest double decycling order processes 10 million nucleotides in less than half a minute for *k* = 100, achieving much lower density than a random order. Miniception is implemented in Python and has a consistent runtime across all values of *k* but is much slower than other methods in two of the three regimes and achieves higher density than MDS-based orders.

The implementation of lookup-based methods for the precomputed MDS-based order and UHS-based orders represents all 4^*k*^ *k*-mers in a boolean vector. In contrast, the memory overhead of the random and MDS-based orders computed on the fly is negligible, and the memory usage is dominated by the sequence being processed. For the lookup-based methods, memory grows exponentially with *k*, and for a 10 million–nt sequence, the memory needed to store the set begins to dominate the memory for the sequence starting at *k* = 14. The memory usage, measured as the maximum resident set size, for *k* = 19 reaches 33.6 GB and is prohibitively large for *k* = 20.

## Discussion

In this study, we developed a method to generate highly effective minimizer orders for any *k*. A major limitation of minimizer orders based on UHSs was the need to create and store the whole set in advance. Instead, we based our new order only on an MDS, avoiding the need to add *k*-mers to make the set universal. This bypasses the costliest steps in DOCKS and PASHA and generates minimizer orders that are even better in terms of their density factors. Furthermore, this approach enables calculating the minimizers in a sequence efficiently on the fly, without the need to store the set. We showed that based on Mykkeltveit's algorithm, we can determine in *O*(*k*) if a *k*-mer belongs to the MDS, and thus, MDS membership can be checked for all *k*-mers in a sequence. The resulting new orders are comparable or better in their density than UHS-based minimizer orders, thus achieving good performance while avoiding escalating runtime and memory usage with the increase of *k*.

In addition, we defined double decycling-set-based minimizer orders. For larger *k* and *L* ≤ 2*k*, the double decycling-set-based orders yielded much lower density than the decycling-set-based orders (Fig. 3) at the cost of a small increase in runtime. As the density of the two methods becomes similar as *L* increases, we recommend using double decycling-set-based orders for *L* ≤ 2.5*k* to achieve lower density, whereas decycling-set-based orders can be used for *L* > 2.5*k* to achieve similar density with slightly faster runtime. When *L* ≫ *k*, our new MDS-based orders achieved only a modest advantage in density over the much faster random order (Fig. 4). We note that the idea of using alternate decycling sets can be extended to using any number of sets, each defined by the first cyclic rotation of a *k*-mer whose embedding crosses any line in the complex plane, not just one of the axes.

We see several promising future directions. Our work focused on general minimizer orders, but other sequence sketches are sequence specific or relax the strict window guarantee of minimizers to obtain improved performance. The advantages of an MDS are likely to extend to these methods. For example, frequency-based orders are known to be highly efficient in terms of density and are easily computable as sequence-specific minimizer orders. It will be interesting to extend our work by ranking each of the sets in a partition by their frequency in a specific sequence data set to achieve lower density values (as was recently shown by incorporating UHS-based orders with frequency ranking [Nyström-Persson et al. 2021]). In addition, it would be possible to use decycling sets and their variants as sketches without defining compatible minimizer orders by simply including all decycling-set *k*-mers in the sketch. Although not providing a window guarantee, such schemes would be better conserved under mutations than minimizers, as they are not dependent on a longer sequence window (Edgar 2021).

There are also open theoretical and technical questions arising from our study. It is necessary to explain why the densities of the MDS-based minimizer orders match the shape of the theoretical lower bound for the more general forward sequence sketching schemes. The gap observed between the MDS-based order and the lower bound should be studied, and hopefully, a tighter lower bound for minimizer schemes can be found. Performance analysis of our new minimizer orders in terms of other criteria, including maximum bin load (Chikhi et al. 2016; Flomin et al. 2022) and conservation (Edgar 2021), is also a worthy goal.

Immediate future work should improve the implementation for *k* > 63 to speed up runtimes for very large *k*. In addition, the implementations using precomputed UHS or MDS could use more efficient data structures to store the set, for example, Bloom filters (Bloom 1970) or prefix tries as in DeBlasio et al. (2019). More efficient data structures would allow for faster lookup-based methods to be extended to larger *k*, but the exponential dependence on *k* in the size of the sets means that a memory bottleneck would be reached very quickly. Such speedups could also make the runtimes of MDS-based minimizer orders for longer *k* more competitive with random minimizer orders. The runtimes of random minimizer orders are extremely fast even for large *k*, resulting in a trade-off between improved density and increased runtimes for our decycling-set-based minimizers.

To conclude, we expect our new approach to enable more efficient analyses of high-throughput sequencing data. By implementing our new MDS-based minimizer orders in data structures and algorithms of high-throughput DNA sequencing analysis, we expect to achieve reductions in runtime and memory beyond what was previously shown using UHS-based minimizer orders.

### Software availability

All code developed under this project is publicly available at GitHub (https://github.com/OrensteinLab/DecyclingSetBasedMinimizerOrder) and as Supplemental Code.

## Supplementary Material

Supplement 1

Supplement 2

## References

[GR277644PELC1] Bloom BH. 1970. Space/time trade-offs in hash coding with allowable errors. Commun ACM 13: 422–426. 10.1145/362686.362692

[GR277644PELC2] Chikhi R, Limasset A, Medvedev P. 2016. Compacting de Bruijn graphs from sequencing data quickly and in low memory. Bioinformatics 32: i201–i208. 10.1093/bioinformatics/btw279 27307618 PMC4908363

[GR277644PELC3] DeBlasio D, Gbosibo F, Kingsford C, Marçais G. 2019. Practical universal *k*-mer sets for minimizer schemes. In Proceedings of the 10th ACM International Conference on Bioinformatics, Computational Biology and Health Informatics, BCB ‘19, pp. 167–176. Association for Computing Machinery, New York.

[GR277644PELC4] Deorowicz S, Kokot M, Grabowski S, Debudaj-Grabysz A. 2015. KMC 2: fast and resource-frugal *k*-mer counting. Bioinformatics 31: 1569–1576. 10.1093/bioinformatics/btv022 25609798

[GR277644PELC5] Dutta A, Pellow D, Shamir R. 2022. Parameterized syncmer schemes improve long-read mapping. PLoS Comput Biol 18: e1010638. 10.1371/journal.pcbi.1010638 36306319 PMC9645665

[GR277644PELC6] Edgar R. 2021. Syncmers are more sensitive than minimizers for selecting conserved *k*-mers in biological sequences. PeerJ 9: e10805. 10.7717/peerj.10805 33604186 PMC7869670

[GR277644PELC7] Ekim B, Berger B, and Orenstein Y. 2020. A randomized parallel algorithm for efficiently finding near-optimal universal hitting sets. In Research in computational molecular biology, pp. 37–53. Springer International Publishing, Cambridge, MA. 10.1007/978-3-030-45257-5_3 PMC11148856 38835399

[GR277644PELC8] Ekim B, Berger B, Chikhi R. 2021. Minimizer-space de Bruijn graphs: whole-genome assembly of long reads in minutes on a personal computer. Cell Syst 12: 958–968.e6. 10.1016/j.cels.2021.08.009 34525345 PMC8562525

[GR277644PELC9] Flomin D, Pellow D, Shamir R. 2022. Data set-adaptive minimizer order reduces memory usage in *k*-mer counting. J Comput Biol 29: 825–838. 10.1089/cmb.2021.0599 35527644

[GR277644PELC10] Fredricksen H, Maiorana J. 1978. Necklaces of beads in *k* colors and *k*-ary de Bruijn sequences. Discrete Math 23: 207–210. 10.1016/0012-365X(78)90002-X

[GR277644PELC11] Hoang M, Zheng H, Kingsford C. 2022. Differentiable learning of sequence-specific minimizer schemes with DeepMinimizer. J Comput Biol 29: 1288–1304. 10.1089/cmb.2022.0275 36095142 PMC9807081

[GR277644PELC12] Holley G, Melsted P. 2020. Bifrost: highly parallel construction and indexing of colored and compacted de Bruijn graphs. Genome Biol 21: 249. 10.1186/s13059-020-02135-8 32943081 PMC7499882

[GR277644PELC13] Karp R. 1972. Reducibility among combinatorial problems. In Complexity of computer computations (ed. R Miller, J Thatcher), pp. 85–103. Plenum Press, Boston, MA.

[GR277644PELC14] Li H. 2018. Minimap2: pairwise alignment for nucleotide sequences. Bioinformatics 34: 3094–3100. 10.1093/bioinformatics/bty191 29750242 PMC6137996

[GR277644PELC15] Marçais G, Pellow D, Bork D, Orenstein Y, Shamir R, Kingsford C. 2017. Improving the performance of minimizers and winnowing schemes. Bioinformatics 33: i110–i117. 10.1093/bioinformatics/btx235 28881970 PMC5870760

[GR277644PELC16] Marçais G, DeBlasio D, Kingsford C. 2018. Asymptotically optimal minimizers schemes. Bioinformatics 34: i13–i22. 10.1093/bioinformatics/bty258 29949995 PMC6037127

[GR277644PELC17] Marchet C, Kerbiriou M, Limasset A. 2021. BLight: efficient exact associative structure for k-mers. Bioinformatics 37: 2858–2865. 10.1093/bioinformatics/btab217 33821954

[GR277644PELC18] Mykkeltveit J. 1972. A proof of Golomb's conjecture for the de Bruijn graph. J Comb Theory B 13: 40–45. 10.1016/0095-8956(72)90006-8

[GR277644PELC19] Nurk S, Koren S, Rhie A, Rautiainen M, Bzikadze AV, Mikheenko A, Vollger MR, Altemose N, Uralsky L, Phillippy AM, 2022. The complete sequence of a human genome. Science 376: 44–53. 10.1126/science.abj6987 35357919 PMC9186530

[GR277644PELC20] Nyström-Persson J, Keeble-Gagnère G, Zawad N. 2021. Compact and evenly distributed *k*-mer binning for genomic sequences. Bioinformatics 37: 2563–2569. 10.1093/bioinformatics/btab156 33693556 PMC8428581

[GR277644PELC21] Orenstein Y, Pellow D, Marçais G, Shamir R, Kingsford C. 2017. Designing small universal *k*-mer hitting sets for improved analysis of high-throughput sequencing. PLoS Comput Biol 13: e1005777. 10.1371/journal.pcbi.1005777 28968408 PMC5645146

[GR277644PELC22] Pibiri GE. 2022. Sparse and skew hashing of K-mers. Bioinformatics 38: i185–i194. 10.1093/bioinformatics/btac245 35758794 PMC9235479

[GR277644PELC23] Rautiainen M, Marschall T. 2021. MBG: Minimizer-based sparse de Bruijn Graph construction. Bioinformatics 37: 2476–2478. 10.1093/bioinformatics/btab004 33475133 PMC8521641

[GR277644PELC24] Sahlin K. 2022. Strobealign: flexible seed size enables ultra-fast and accurate read alignment. Genome Biol 23: 260. 10.1186/s13059-022-02831-7 36522758 PMC9753264

[GR277644PELC25] Schleimer S, Wilkerson DS, Aiken A. 2003. Winnowing: local algorithms for document fingerprinting. In Proceedings of the 2003 ACM SIGMOD International Conference on Management of Data, San Diego, CA, pp. 76–85.

[GR277644PELC26] Wood DE, Salzberg SL. 2014. Kraken: ultrafast metagenomic sequence classification using exact alignments. Genome Biol 15: R46. 10.1186/gb-2014-15-3-r46 24580807 PMC4053813

[GR277644PELC27] Zheng H, Kingsford C, and Marçais G. 2020a. Lower density selection schemes via small universal hitting sets with short remaining path length. In Research in computational molecular biology, pp. 202–217. Springer International Publishing, Berlin, Heidelberg, Germany. 10.1089/cmb.2020.0432 PMC8066347 33325773

[GR277644PELC28] Zheng H, Kingsford C, Marçais G. 2020b. Improved design and analysis of practical minimizers. Bioinformatics 36: i119–i127. 10.1093/bioinformatics/btaa472 32657376 PMC8248892

[GR277644PELC29] Zheng H, Kingsford C, Marçais G. 2021. Sequence-specific minimizers via polar sets. Bioinformatics 37: i187–i195. 10.1093/bioinformatics/btab313 34252928 PMC8686682

