# Supplementary material for: Efficient minimizer orders for large values of k using minimum decycling sets
Source: Genome Res. 2023 Jul;33(7):1154–61. doi: 10.1101/gr.277644.123 (PMC10538483; doi:10.1101/gr.277644.123)
Supplement: Supplement 2 [file Supplemental_Material.pdf]

Supplementary information for: Efficient minimizer orders for large values of k using minimum decycling sets

David Pellow[1], Lianrong Pu[1], Baris Ekim[2], Lior Kotlar[3], Bonnie Berger[2,4], Ron Shamir[1]*, and Yaron Orenstein[5,6]*

[1] Blavatnik School of Computer Science, Tel-Aviv University, Israel
[2] Computer Science and Artificial Intelligence Laboratory, Massachusetts Institute of Technology, Cambridge, MA, USA
[3] Department of Computer Science, Ben-Gurion University, Israel
[4] Department of Mathematics, Massachusetts Institute of Technology, Cambridge, MA, USA
[5] Department of Computer Science, Bar-Ilan University, Israel
[6] The Mina and Everard Goodman Faculty of Life Sciences, Bar-Ilan University, Israel

Supplementary results are shown in Supplementary Figures S1 and S2. In Figure S1 particular densities are computed over 10 runs on different 10M nt samples from chromosome X of the CHM13 assembly and expected densities are computed on 10 random 10M nt sequences, each run using a different random seed for the hash function. In Figure S2, panels A,D,G,J, and M show the particular density for a single run over the entire 154M nt sequence of chromosome X. The other panels show particular density across 10 runs with different random seeds for the whole genomes of *E. coli* and *Klebsiella pneumoniae* bacteria.

* shared correspondence

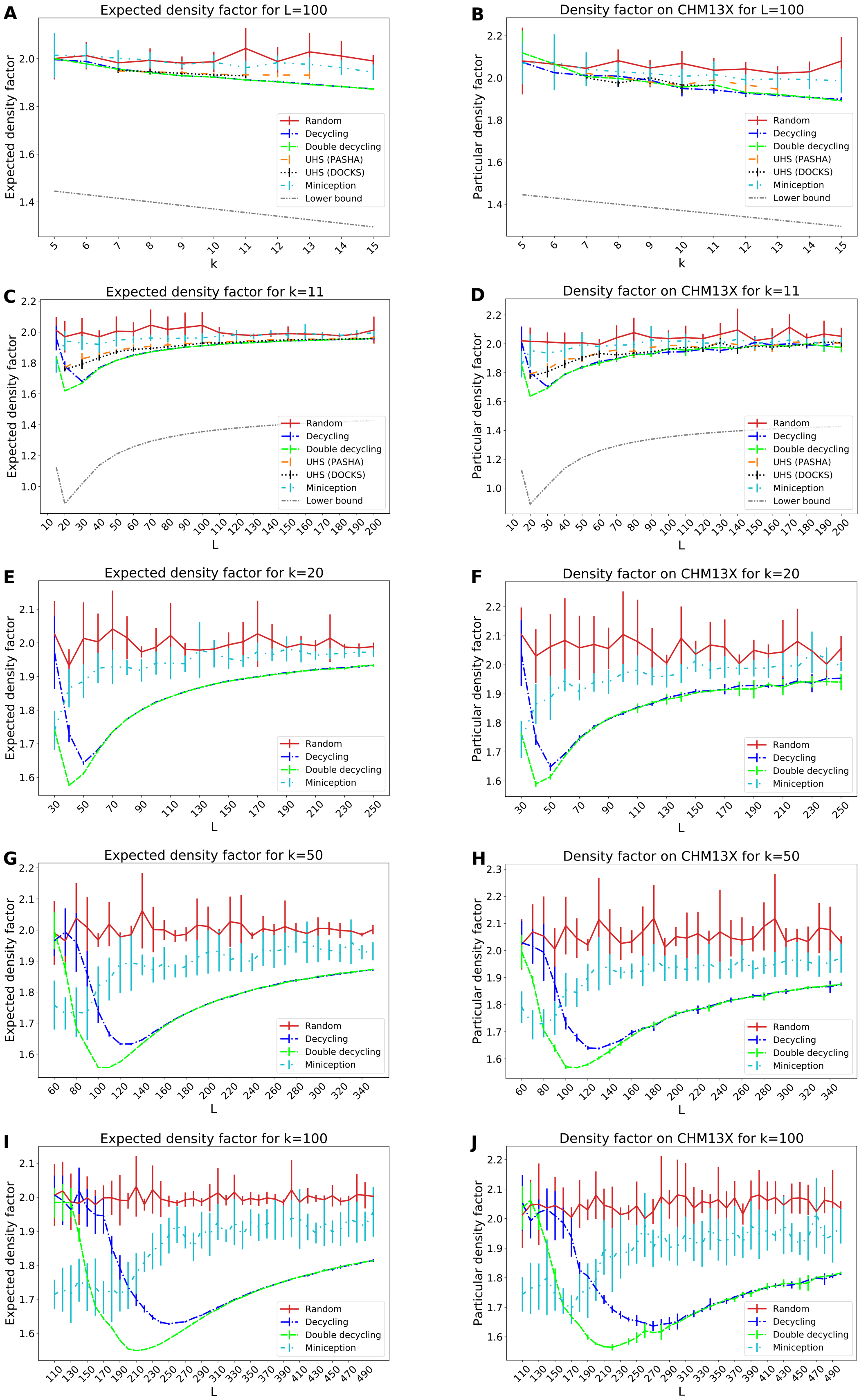

Fig. S1: **Density factor of various minimizer orders including error bars.** The expected density (left) and particular density factors on samples from CHM13X (right) of different minimizer orders is compared over $L = 100$ and $5 \leq k \leq 15$ (**A,B**) and for a range of fixed k with varying L (**C-J**). The averages and standard deviations over 10 runs with 10M nt sequences are shown.

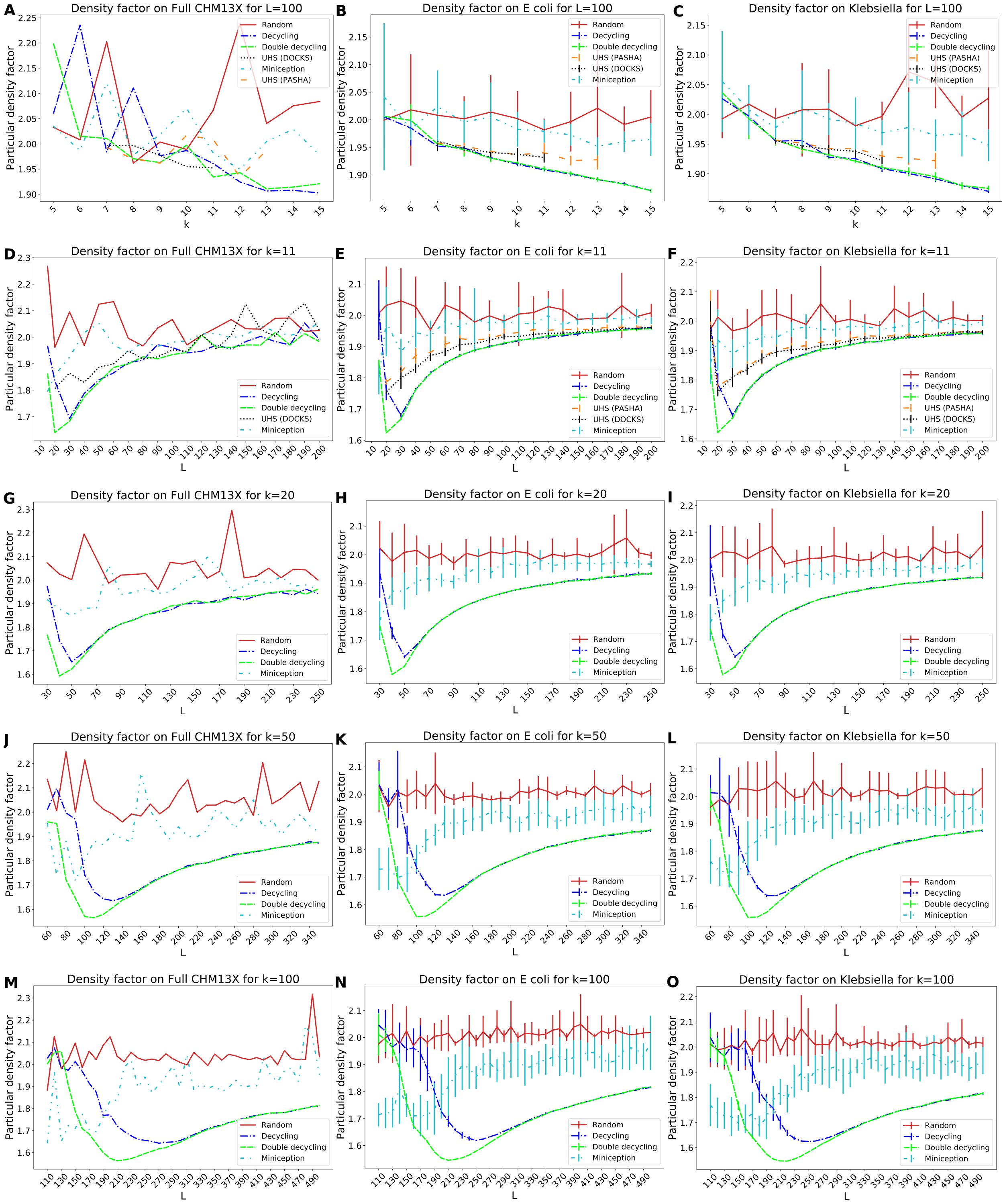

Fig. S2: **Particular density factors of various minimizer orders over genome sequences.** The particular density factors are shown for: the entire 154M nt sequence of chromosome X from CHM13 (**left**); the 4.9M nt genome of *E. coli* strain W (RefSeq accession GCF_000184185.1) (**center**); and the 5.3M nt genome of *Klebsiella pneumoniae* strain HS11286 (RefSeq accession GCF_000240185.1) (**right**). For the bacterial genomes, averages and standard deviations over 10 runs are shown.
